# Functionally Active Fc Mutant Antibodies Recognizing Cancer Antigens Generated Rapidly at High Yields

**DOI:** 10.3389/fimmu.2017.01112

**Published:** 2017-09-11

**Authors:** Kristina M. Ilieva, Judit Fazekas-Singer, Daniela Y. Achkova, Tihomir S. Dodev, Silvia Mele, Silvia Crescioli, Heather J. Bax, Anthony Cheung, Panagiotis Karagiannis, Isabel Correa, Mariangela Figini, Rebecca Marlow, Debra H. Josephs, Andrew J. Beavil, John Maher, James F. Spicer, Erika Jensen-Jarolim, Andrew N. Tutt, Sophia N. Karagiannis

**Affiliations:** ^1^St John’s Institute of Dermatology, Division of Genetics and Molecular Medicine, King’s College London, Guy’s Hospital, London, United Kingdom; ^2^Breast Cancer Now Unit, School of Cancer Sciences, Guy’s Cancer Centre, King’s College London, London, United Kingdom; ^3^Comparative Medicine, The Interuniversity Messerli Research Institute of the University of Veterinary Medicine Vienna, University of Vienna, Vienna, Austria; ^4^Institute of Pathophysiology and Allergy Research, Center of Pathophysiology, Infectiology and Immunology, Medical University of Vienna, Vienna, Austria; ^5^School of Cancer Sciences, King’s College London, Bermondsey Wing, Guy’s Hospital, London, United Kingdom; ^6^Randall Division of Cell and Molecular Biophysics, King’s College London, New Hunt’s House, London, United Kingdom; ^7^Department of Oncology, Haematology and Stem Cell Transplantation, University Hospital of Hamburg Eppendorf, Hamburg, Germany; ^8^Molecular Therapies Unit, Department of Experimental Oncology and Molecular Medicine, Fondazione IRCCS Istituto Nazionale dei Tumori Milano, Milan, Italy; ^9^Department of Clinical Immunology and Allergy, King’s College Hospital NHS Foundation Trust, London, United Kingdom; ^10^Department of Immunology, Eastbourne Hospital, Eastbourne, United Kingdom

**Keywords:** antibodies, cancer immunotherapy, mutants, cloning, expression, ADCC, HER2, chondroitin sulfate proteoglycan 4

## Abstract

Monoclonal antibodies find broad application as therapy for various types of cancer by employing multiple mechanisms of action against tumors. Manipulating the Fc-mediated functions of antibodies that engage immune effector cells, such as NK cells, represents a strategy to influence effector cell activation and to enhance antibody potency and potentially efficacy. We developed a novel approach to generate and ascertain the functional attributes of Fc mutant monoclonal antibodies. This entailed coupling single expression vector (pVitro1) antibody cloning, using polymerase incomplete primer extension (PIPE) polymerase chain reaction, together with simultaneous Fc region point mutagenesis and high yield transient expression in human mammalian cells. Employing this, we engineered wild type, low (N297Q, NQ), and high (S239D/I332E, DE) FcR-binding Fc mutant monoclonal antibody panels recognizing two cancer antigens, HER2/*neu* and chondroitin sulfate proteoglycan 4. Antibodies were generated with universal mutagenic primers applicable to any IgG1 pVitro1 constructs, with high mutagenesis and transfection efficiency, in small culture volumes, at high yields and within 12 days from design to purified material. Antibody variants conserved their Fab-mediated recognition of target antigens and their direct anti-proliferative effects against cancer cells. Fc mutations had a significant impact on antibody interactions with Fc receptors (FcRs) on human NK cells, and consequently on the potency of NK cell activation, quantified by immune complex-mediated calcium mobilization and by antibody-dependent cellular cytotoxicity (ADCC) of tumor cells. This strategy for manipulation and testing of Fc region engagement with cognate FcRs can facilitate the design of antibodies with defined effector functions and potentially enhanced efficacy against tumor cells.

## Introduction

Monoclonal antibodies for cancer therapy engender a range of anti-tumor functions. These may be defined by Fab region recognition of target antigen epitopes and by engagement of the Fc region with cognate Fc receptors (FcR) on a variety of effector cell subsets. Anti-tumor functions can include direct tumor cell killing through Fab-mediated antagonistic activity (1–6), or modulating the behavior of cytotoxic T cells by checkpoint blockade (7, 8). Furthermore, Fc-mediated effects such as complement-dependent cytotoxicity (CDC) (6, 9, 10), antibody-dependent cellular cytotoxicity (ADCC) (5, 6, 11, 12) and phagocytosis (ADCP) (13) constitute important functions of a large proportion of clinically used antibodies in oncology. As many antibodies can exert multimodal anti-tumor activities influenced by both Fab- and Fc-mediated effects (6, 14), engineering antibodies with defined attributes and delineating their mechanisms of action is important for the generation of effective therapeutic agents.

The Fc portion of the antibody and the nature and affinity of its interactions with FcRs are critical in determining antibody serum and tissue half-life, bio-distribution (15), recruitment, and activation of immune effector cells such as NK cells and macrophages against specific target cells (16). Even though monoclonal antibodies are potent anti-cancer therapeutics, their potency in restricting disease progression may be limited by low affinity for activating FcRs, competition with endogenous serum antibodies, low half-life in tissues and interactions with the inhibitory Fcγ receptor, FcγRIIB, which restricts effector cell functions (17, 18). On the other hand, agonistic antibodies specific for the TNFR family molecules have been reported to be significantly more efficacious if they have higher affinity for the inhibitory receptor FcγRIIB and lower affinity for activating FcRs (19, 20). This further highlights the crucial importance of assessing the contributions of the Fc region in different antibody immunotherapy approaches for cancer and the unmet need for novel Fc-engineered antibody variants to serve the purposes of specific strategies. Protein engineering methods utilizing structural bioinformatics and computational design have identified amino acid residues that can play key roles in enhancing antibody FcR-binding and Fc-mediated functional capabilities (21). Alternatively, abrogation of antibody binding to FcRs and abolition of Fc-mediated effector functions could be a useful approach for the design of antibodies with direct agonistic or antagonistic activities where effector cell engagement may not be desirable (22–25). Developing a strategy to easily manipulate antibody Fc regions can help to dissect the mechanisms of action of anti-cancer antibodies, allowing analysis of Fab-mediated functions in the presence or absence of Fc-mediated activities. Such approaches could find a broad applicability in therapeutic antibody engineering and translation (26–28).

*In vitro* and *in vivo* potency and mechanistic evaluations of engineered antibodies and their downstream applications in cancer research are heavily dependent on the availability of sufficient quantities of high quality functional material generated from expression systems such as human embryonic kidney (HEK293), Chinese hamster ovary (CHO), and mouse myeloma (SP2/0, NS0) cells (29–31), mostly utilizing variable regions derived from hybridoma (32, 33) or phage display technologies (34). Current approaches largely rely on the generation of stable expressing cell lines, and do not include efficient built-in tools for sequence engineering and mutagenesis, which may be lengthy and labor-intensive (35).

We previously reported the design and implementation of a single dual expression vector system combined with efficient insertion of any antibody variable and constant regions through polymerase incomplete primer extension (PIPE) cloning. We showed that this can facilitate antibody production by human embryonic kidney (HEK293F) cells (36, 37). In this study, by employing a novel cloning approach based on PIPE combined with simultaneous point mutagenesis, we generate monoclonal antibodies specific for tumor-associated antigens with modified Fc domains designed to alter interactions with immune effector cells. Most well established mutagenesis cloning methods require a two round PCR method or cannot be applied to large plasmids without increasing the risk of random amplification error (37–40). Our study represents an improvement of traditional PCR mutagenesis methods by offering efficient mutagenesis (requiring one round of PCR only), combined with enzyme-free cloning for the generation of large expression-ready constructs (over 8,000 kb). We also designed this system to allow generation of different versions of the same antibody construct. This could find wide applicability for functional and translational studies and could be applied to any IgG1 antibody due to the universal nature of the mutagenesis approach we are employing. To our knowledge, this is the first antibody production platform that combines generation and functional validation of high yields of specific Fc mutant antibodies. With this strategy, we aim to design agents with defined effector functions in a substantially shorter timeframe, employing small culture volumes and at significantly higher yields.

## Materials and Methods

### Isolation of Human Immune Cells

Peripheral blood was obtained through the UK National Health System (NHS) Blood and Transplant system from anonymous donor leukocyte cones. NK cells were isolated using RosetteSep™ Human NK Cell Enrichment Cocktail (STEMCELL™ Technologies), according to the manufacturer’s instructions.

### Cell Culture

All tumor cell lines were sustained at 37°C in a humidified atmosphere in 5% CO_2_, unless otherwise specified. Cell culture medium was supplemented with 10% fetal calf serum (FCS, Thermo Fischer Scientific), unless otherwise specified. Adherent cells were detached using 0.25% Trypsin-EDTA except for cancer cell lines expressing the trypsin sensitive antigen chondroitin sulfate proteoglycan 4 (CSPG4), which were detached using 5 mM EDTA solution in phosphate buffered saline (PBS). The cell lines BT-474 (invasive ductal carcinoma, primary site derived), SK-BR-3 (invasive ductal carcinoma, metastasis origin) MDA-MB-231 (invasive ductal carcinoma, metastasis origin), and Hs 578T (breast carcinoma, primary site derived) were purchased from the American Tissue Culture Collection (ATCC) and cultured in DMEM GlutaMAX™ (Thermo Fischer Scientific). The cell lines HCC1954 (invasive ductal carcinoma, primary site derived), MDA-MB-231 HTB-26 (human breast adenocarcinoma, metastasis origin), ZR-75-30 (invasive ductal carcinoma, metastasis origin) and BT-549 (invasive ductal carcinoma, lymph node metastasis origin) were purchased from ATCC and cultured in RPMI GlutaMAX™ (Thermo Fischer Scientific). MDA-MB-231-CSPG4^+++^ cells were generated in-house by knocking in the coding sequence of the full-length tumor-associated antigen CSPG4 (GenBank accession number BC172576) and cells were cultured as the wild type (WT) cells. Expi293F cells (Thermo Fischer Scientific) were grown on a Stuart orbital shaker (model SSL1) (41), at 125 rpm in 8% CO_2_ in serum-free Expi293 expression medium (Thermo Fischer Scientific).

### Generation of Fc Mutant Antibodies

The amino acid sequences of the trastuzumab heavy and light variable regions were obtained from the DrugBank database (www.drugbank.ca), translated in nucleotide sequences and manually codon optimized for a human expression host. Optimized sequences were synthesized using GeneArt Gene Synthesis (Thermo Fischer Scientific). The variable region fragments of trastuzumab were then cloned into pVitro1-hygro-mcs vector as previously described (36). PIPE PCR was then performed using the ready cloned WT antibody pVitro1 constructs as a template and mutagenic PIPE primers to generate four linear fragments of the construct with 5′ PIPE overhangs (“sticky ends” for PIPE cloning) and the desired point mutations (Table 1). The PCR reagents are listed in Table S1 in Supplementary Material. The cycling conditions used for execution of the mutagenic PCRs were as described before (36), but with varying extension times (Table 2), depending on the length of the fragment, optimized to favor the incomplete extension. The PCRs were performed on a ProFlex 3 × 32-well PCR System thermal cycler (Thermo Fischer Scientific). The amplified DNA fragments were subjected to DpnI (New England Biolabs) digestion for 2 h at 37°C. Following the digestion, the four PCR products were diluted three times with deionized water, then mixed unpurified in a ratio of 1:1:1:1, incubated at room temperature for 30 min to overnight and between 4 and 10 µL of the mixture were transformed into One Shot TOP10 chemically competent *E. coli* cells (Thermo Fischer Scientific), according to the manufacturer’s instructions. Successful cloning was confirmed by Sanger sequencing (Source BioScience Sanger Sequencing Service, UK).

**Table 1:** PCR primers for generation of anti-HER2 and anti–CSPG4 Fc mutant antibodies.

Primer name	Sequence 5′→3′	Mutation	Amplified fragment
F-DE-1	CAGCACCTGAACTCCTGGGGGGACCG 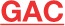 GTCTTCCTCTTCCCCC	S239D	DE F1
R-DE-1	GGGGCTGCCCTTTGGCTTTGGAGATGGTTTTCTC 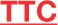 GGGGGCTGG	I332E	DE F1
F-DE-2	CCAGCCCCC 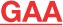 GAGAAAACCATCTCCAAAGCCAAAGGGCAGCCCC	I332E	DE F2
R-pVitro-1-Kappa	ACCGCGGCTAGCTGGAACCCAGAGCAGCAGAAACCCAATG	–	DE F2
F-pVitro-VL-Univ	CATTGGGTTTCTGCTGCTCTGGGTTCCAGCTAGCCGCGGT	–	DE F3
R-DE-3/R-NQ-3	GGGGGAAGAGGAAGACGTCCGGTCCCCCCAGGAGTTCAGGTGCTG	–	DE F3; NQ F3
F-DE-4/F-NQ-4	GTTGCTTTGATTACAACACTGGAGAGAAATGCAGCATGTTGCTGATT	–-	DE F4; NQ F4
R-DE-4	GGGGGAAGAGGAAGAC 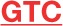 CGGTCCCCCCAGGAGTTCAGGTGCTG	S239D	DE F4
F-NQ-1	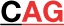 AGCACGTACCGGGTGGTCAGCGTCCTCACCGTCCTGCACCAG	N297Q	NQ F1
R-NQ-1	TGATCTACCCGCGCTCAGCCCTGGGCGCATGCTCCTCGCGCTGTC	–	NQ F1
F-NQ-2	CGAGGAGCATGCGCCCAGGGCTGAGCGCGGGTAGATCAGAGCACA	–	NQ F2
R-NQ-2	TACAAAGTGTTACCCCTCTAGACCTGGAAAGACCAGGCGGAGTTT	–	NQ F2
F-NQ-3	GCCTGGTCTTTCCAGGTCTAGAGGGGTAACACTTTGTACTGCGTT	–	NQ F3
R-NQ-4	AGGACGGTGAGGACGCTGACCACCCGGTACGTGCT 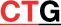 GTACTGC	N297Q	NQ F4

*Underlined nucleotides depict mutated codons; sequences in red font indicate mutated nucleotides*.

**Table 2:** PIPE PCR fragments with accompanying specific PCR extension times for fragment amplification.

Fragment	Approximate size (bp)	Extension time (s)
NQ F1	~2,000	28
NQ F2	~2,000	28
NQ F3	~2,000	28
NQ F4	~2,600	35
DE F1	~350	5
DE F2	~3,150	42
DE F3	~2,800	35
DE F4	~2,400	35

### Production of Monoclonal Antibodies

Expi293F cells were transfected with the pVitro1-hygro-mcs antibody construct using the ExpiFectamine293 Transfection kit (Thermo Fischer Scientific) as per manufacturer’s instructions. Transfection was carried out in Expi293 Expression Medium (Thermo Fischer Scientific). Supernatants of Expi293F cells transfected with an indicated antibody construct were harvested, spun down at 300 × *g* for 5 min and then at 3,100 × *g* for 40 min and filtered using a 0.45 µm membrane. For comparative studies, anti-CSPG4 WT IgG1 antibody (anti-CSPG4 WT) was produced using the previously described HEK293-F method (36), as follows: starting with transient transfection and using 30 mL cultures in 125 mL shaker flasks for 2 weeks under Hygromycin B (Thermo Fischer Scientific) selection, those were subsequently scaled up to 500 mL in 1 L shaker flasks. The supernatants were harvested after 2 weeks of stable antibody expression without antibiotic selection. Monoclonal antibodies were purified using Pierce™ Protein A Columns, 1 mL (Thermo Fischer Scientific). Antibodies were eluted using acetate elution buffer (0.58% glacial acetic acid, 0.15 M Sodium chloride) and neutralized with 120 µL neutralization buffer (1 M Tris, pH = 9). Purified antibodies were stored in PBS.

### Aleuria Aurantia Lectin (AAL) Western Blot

Purified antibody samples (500 ng) were mixed with 4× Laemmli buffer (Bio-Rad) containing 10% 2-mercaptoethanol and incubated at 95°C for 10 min. Subsequently, the reduced samples were run on 4–15% precast polyacrylamide gels (Bio-Rad) and semi-dry blotted using the Bio-Rad Trans-Blot^®^ Turbo™ Blotting System, according to the manufacturer’s instruction. The membrane was then cut into two just above 35 kDa to separate the antibody heavy chain (50 kDa) and light chain (25 kDa), using the PageRuler prestained protein ladder (Thermo Scientific) as a reference. Next, the membrane containing proteins above 35 kDa was blocked using Carbo-Free™ Blocking Solution (Vectorlab) and the one containing proteins below 35 kDa—in 5% bovine serum albumin (BSA) solution in PBS 0.05% Tween 20 (PBST). For probing, the over-35 kDa membrane was incubated with 0.2 µg/mL biotinylated AAL in Carbo-Free™ Blocking Solution for 1 h at room temperature and subsequently washed in PBST 3× for 10 min. Next, the membrane was incubated with horseradish peroxidase (HRP)-conjugated streptavidin (Thermo Fischer Scientific, 1:30,000) for one hour, washed and developed with Enhanced Chemiluminescence (ECL, GE Healthcare). The under-35 kDa membrane was probed with rabbit anti-human-kappa light chain antibody (Abcam, 1:4,000) in PBST 5% BSA for 1 h at room temperature, washed and incubated with anti-rabbit-IgG HRP antibody (Cell Signaling Technology, 1:2,000), washed again, and developed as above. The results were analyzed using the ImageJ software.

### Western Blot Semi-Quantitative Analysis

The AAL signal values of the different protein bands (peak area) were normalized to the anti-kappa signal values (LC kappa). The AAL/LC kappa ratio values of anti-HER2 antibody variants were presented as a proportion (percentage) of the value of trastuzumab. Similarly, anti-CSPG4 antibody variant values were presented as a proportion of anti-CSPG4 WT.

### Evaluations of Antibody Yields by ELISA

IgG antibody expression by Expi293F cells was monitored using a sandwich enzyme-linked immunosorbent assay (ELISA). Briefly, Maxisorp 96-well plates (Thermo Fischer Scientific) were coated overnight with 0.5 µg/mL mouse anti-human IgG1 antibody (Bio-Rad) in carbonate buffer. On the next day, the plate was washed with PBS 0.5% Tween 20 (PBST) and unspecific binding was blocked using 2% skim milk in PBST (blocking buffer). Trastuzumab (Herceptin^®^, Roche) was used at as a standard at a starting concentration of 3 µg/mL. All samples were diluted (1:1,000) in blocking buffer and incubated on the plate overnight. The plates were developed on the next day, following an incubation with anti-human IgG-HRP (Sigma) at a dilution of 1:5,000, using o-Phenylenediamine dihydrochloride (Sigma) as the HRP substrate. Optical density was measured using Fluostar^®^ Omega Spectrophotometer (BMG Labtech), at 492 and 650 nm, the latter used to subtract background absorbance. Standard curves were constructed on the Fluostar^®^ Omega analysis software using a 4-parameter fit.

### Assessment of Antibody Binding to Cells by Flow Cytometry

Recognition of FcγRs on fresh peripheral blood NK cells and of the target antigen on tumor cells by different anti-HER2 and anti-CSPG4 antibody variants were assessed through flow cytometric binding assays. Cells were detached, as described above, re-suspended in staining buffer (PBS, 2% FCS) and incubated in 96-well round-bottom plates (0.2 × 10^6^ cells per well) on ice in the presence of the serially diluted antibody variants (concentration range 0.008–5 µg/mL) for 30 min on ice. The cells were subsequently washed and incubated with 1 µg per test FITC-conjugated goat anti-human IgG (Vector Laboratories Ltd.) on ice, for another 30 min. After one wash, cells were analyzed on a BD LSRFortessa™ (BD Biosciences), using the High Throughput Sampler (HTS) option. The BD Cytofix/Cytoperm™ (Becton Dickinson) kit was used according to the manufacturer’s instructions for Expi293F cell permeabilization and intracellular staining to detect antibody production. The results were analyzed using FlowJo software 7.6.5.

### Cell Proliferation Assay

To determine the effect of anti-HER2 antibody variants on cell proliferation, 5,000 (BT-474) or 1,000 (SK-BR-3, MDA-MB-231, HCC1954) cells per well were plated in a 96-cell well tissue culture plates in complete medium and treated with the serially diluted antibodies (concentration ranges 0.0016–25 µg/mL). Cell proliferation was assessed after 96 (BT474, MDA-MB-231) or 120 h (SK-BR-3, HCC1954), using the CellTiter 96^®^ Aqueous One Solution Cell Proliferation Assay kit (Promega), according to the manufacturer’s instructions. Cell densities were measured using Fluostar^®^ Omega Spectrophotometer (BMG Labtech).

### Antibody-Dependent Cellular Cytotoxicity (ADCC) Assay

ADCC assays were performed using the HER2-overexpressing BT-474 cells as target cells and fresh peripheral blood NK cells as effector cells. NK cells and cancer cells were mixed at E:T ratio 10:1 (0.2 × 10^6^ NK cells: 0.02 × 10^6^ target cells) in RPMI GlutaMAX™ (Thermo Fischer Scientific) containing 2% FCS (Thermo Fischer Scientific). They were incubated in 96-well round bottom plates for 4 h (Thermo Fischer Scientific) in the presence of serially diluted anti-HER2 antibody variants or isotype control antibodies (at concentrations ranging from 64 pg/mL to 5 µg/mL). Cellular cytotoxicity was assessed using the Pierce LDH Cytotoxicity Assay Kit (Thermo Fischer Scientific), according to the manufacturer’s instructions. Absorbance read out was measured at 490 and 680 nm using Fluostar^®^ Omega Spectrophotometer (BMG Labtech) and % cytotoxicity was calculated as per manufacturer’s instructions using the following formula:

$$\%\text{ Cytotoxicity}=\left(\frac{\text{Experimental value}-\text{Effector Cells Spontaneous Control}-\text{Target Cells}}{\text{Target Cell Maximum Lysis Control}-\text{Target Cells Spontaneous Control}}\right)\times \text{100}$$

Half maximal effective concentration (EC50) values at which different anti-HER2 Fc variant antibodies induced ADCC were calculated using the GraphPad Prism software (version 6, GraphPad Software Inc., USA). The data were normalized to a maximal (detergent induced) and minimal (isotype control) cell lysis and analyzed using a non-linear regression model.

### Calcium Flux Assay

Freshly isolated human NK cells were incubated with 1 nM Indo-1 calcium indicator (Thermo Fischer Scientific) for 30 min at 37°C in serum-free medium and subsequently washed and rested for 30 min in complete medium (42). Cells (1 × 10^6^ cells per test) were incubated with anti-HER2 and anti-CSPG4 antibody variants at 15 µg/mL for 30 min at 37°C, as above, and re-suspended in 500 µL RPMI GlutaMAX™ (Thermo Fischer Scientific). Before acquisition each sample was warmed up to 37°C for exactly 5 min. The sample was acquired for 30 s to measure basal fluorescence, then the FcRs on the surface of the NK cells were crosslinked by adding 5 µg goat anti-human IgG F(ab′)2 (Thermo Fischer Scientific). Each sample was acquired for 5 min on BD LSRFortessa™ (BD Biosciences) with the violet laser (405 nm) turned off to avoid interference. Ionomycin at 1 µg/mL was used as a positive control. Results were analyzed using FlowJo software version 8.7.

### Human Specimens

Blood cone samples from anonymized donors were purchased from the National Health Service Blood and Transplant Service, United Kingdom. Sample processing was supported through a local ethical framework conducted in accordance with the Helsinki Declaration and approved by the NHS Research Ethics Committee, Guy’s and St. Thomas’ NHS Trust (“Immunopathogenesis and Molecular Biology in Breast Cancer Subtypes,” REC reference number: 13-LO-1248).

## Results

### Antibody Fc Region Manipulation Design

First, we devised a cloning strategy that allows the manipulation of monoclonal antibody Fc regions in order to influence FcRs binding properties and consequently antibody effector functions. Alongside WT equivalents, we generated Fc-mutant variants designed either to have diminished or enhanced binding to FcRs on human immune effector cells, and we exemplified this strategy by engineering panels of antibody clones that recognize two cancer-associated antigens.

**Figure 1:**
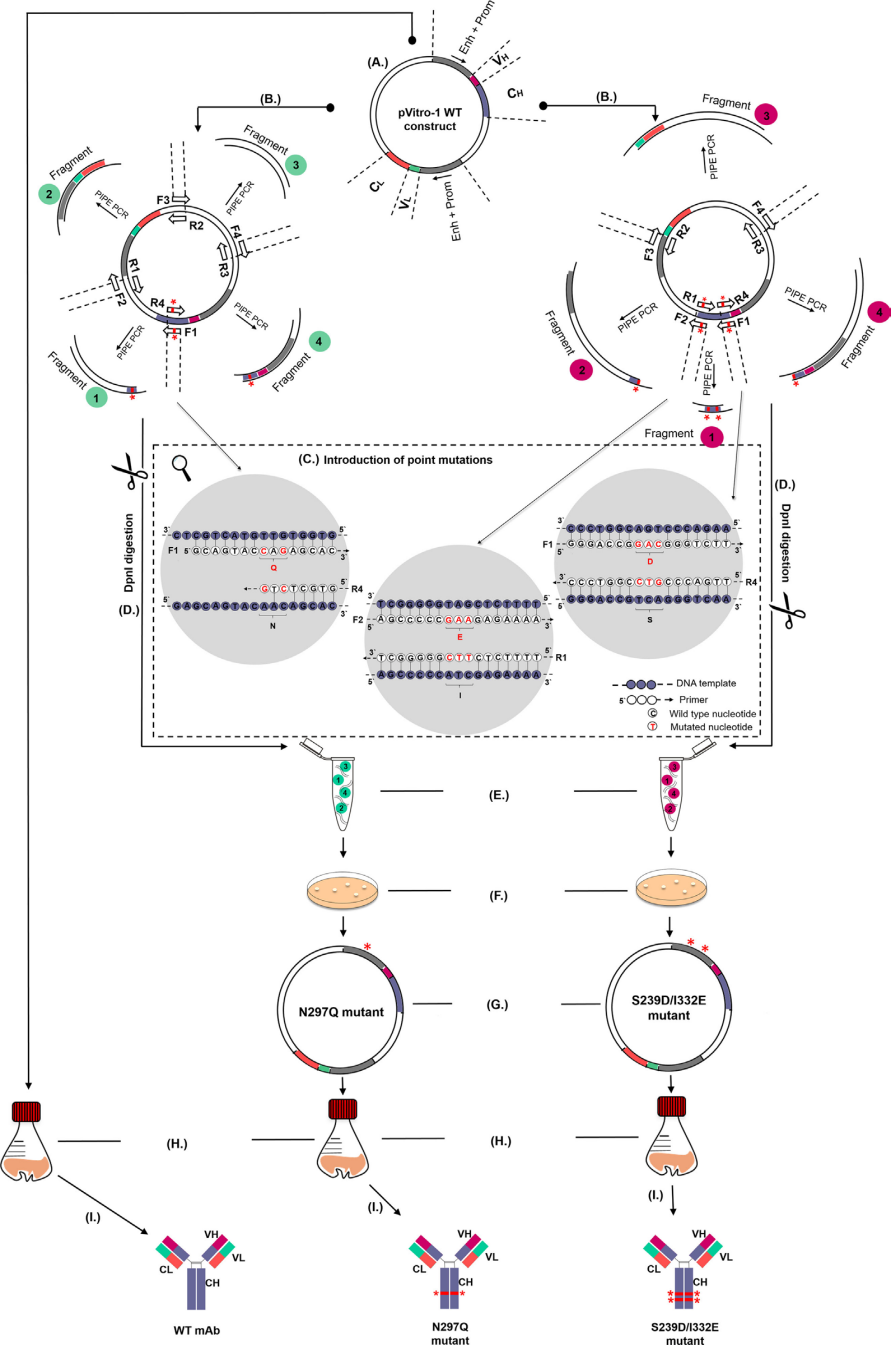
Schematic representation of the pipeline for generation and production of wild-type (WT) and Fc mutant IgG antibodies. **(A)** WT antibody construct in pVitro1-hygro-mcs. **(B)** Polymerase incomplete primer extension (PIPE) PCR linearization and mutagenesis of the WT construct to generate pVitro1 DNA fragments carrying the N297Q (left, fragments 1 and 4) or S239D/I332E (right, fragments 1, 2, and 4) mutations. Mutations indicated by “*”. **(C)** Introduction of mutations in WT constructs through mutagenic PIPE primers. **(D)** DpnI digestion. **(E)** Enzyme-independent assembly of the linear pVitro1 fragments. **(F)** Bacterial transformation of the assembled constructs. **(G)** Confirmation of the insertion of desired mutations. **(H,I)** Recombinant expression in Expi293F cells **(H)** and purification **(I)** of antibody WT and mutant variants.

We initially employed WT IgG1 antibody heavy and light chain fragments incorporated in pVitro1-hygro-mcs vectors (Figure 1A). We then utilized these vectors as templates in four separate PIPE PCRs in order to generate linear fragments carrying specific Fc region point mutations (Figures 1B,C). Point mutations were introduced using mutagenic PIPE primers (Table 1; Table S1 in Supplementary Material). Mutations were designed to either reduce [N297Q (NQ), Figure 1B] or enhance [S239D/I332E (DE), Figure 1C] binding of the antibody Fc to FcγRs (Fc gamma receptors) on human effector cells. Since Asn297 is a conserved glycosylation site for the human IgG1 Fc domain, we mutated this to Gln. This is expected to lead to aglycosylation and a conformational change which can impair binding to FcγRs (26, 28). By contrast, we designed S239D/I332E (DE) mutated IgG1 Fc regions to have increased binding to the FcγRIII compared to WT IgG1 antibodies.

To generate the N297Q mutant antibody variants designed to have reduced binding to FcRs (Figure 1B), we linearized the pVitro1 WT constructs through PIPE PCR (see Table 1 for primer pairs), yielding Fragments 1, 2, 3, and 4. Fragment 1 was generated to carry CAG (Q) instead of AAC (N) at positions 889–891. Shortened extension times and lack of final extension step favored the PIPE process during the PCR. Therefore, the Fragment 1 final product had 5′ PIPE overhangs (“sticky ends”) but also carried the desired CAG codon. Similarly, we generated Fragment 4 with 5′ overhangs and the desired mutated codon. The other two fragments, Fragment 2 and Fragment 3, were as well amplified with 5′ PIPE overhangs but without point mutations.

The S239D/I332E mutants designed to yield antibodies with enhanced FcR binding were generated in a similar manner (see Table 1 for primer pairs). The pVitro1 WT constructs were linearized through PIPE PCR to favor the occurrence of sticky ends for PIPE cloning (Figure 1C). All fragments were amplified with PIPE 5′ overhangs; however, Fragment 1 carried both the GAC (D) codon instead of the WT TCA (S) codon, and the GAA (E) codon instead of the WT ATC (I) codon (positions 877–879 and 994–996, respectively); Fragment 2 carried the GAA (E) codon instead of ATC (I) codon; and Fragment 4 carried GAC (D) codon instead of TCA (S) codon.

The primers used for the generation of both N297Q (NQ) and S239D/I332E (DE) Fc mutants are designed to be universal and can be used for the generation of NQ and DE mutant versions of any IgG1 antibody cloned into pVitro1-hygro-mcs (see Table 2 for lengths of the pVitro1 PIPE fragments and extension times for amplifications).

Following amplification, linear pVitro1 fragments were treated with DpnI enzyme to digest any remnants of the pVitro1 WT construct used as a template in the PCR (Figure 1D). All four fragments were then mixed together unpurified, thus eliminating the need to perform PCR clean up or to use ligation enzymes. The mixtures were incubated at room temperature (Figure 1E) and transformed into competent bacteria (Figure 1F). Correct transformants were verified through Sanger sequencing (Figure 1G). Expi293F cells were transfected with the pVitro1 mutant constructs in 30 mL working volumes (Figure 1H) and antibodies were secreted in culture supernatants (Figure 1I).

Overall, this strategy facilitates the manipulation of monoclonal antibody Fc regions to allow the generation of mutant versions of any IgG1 antibody clone.

### Generation of Anti-HER2 and Anti-CSPG4 N297Q and S293D/I332E IgG1 Mutant Constructs

**Figure 2:**
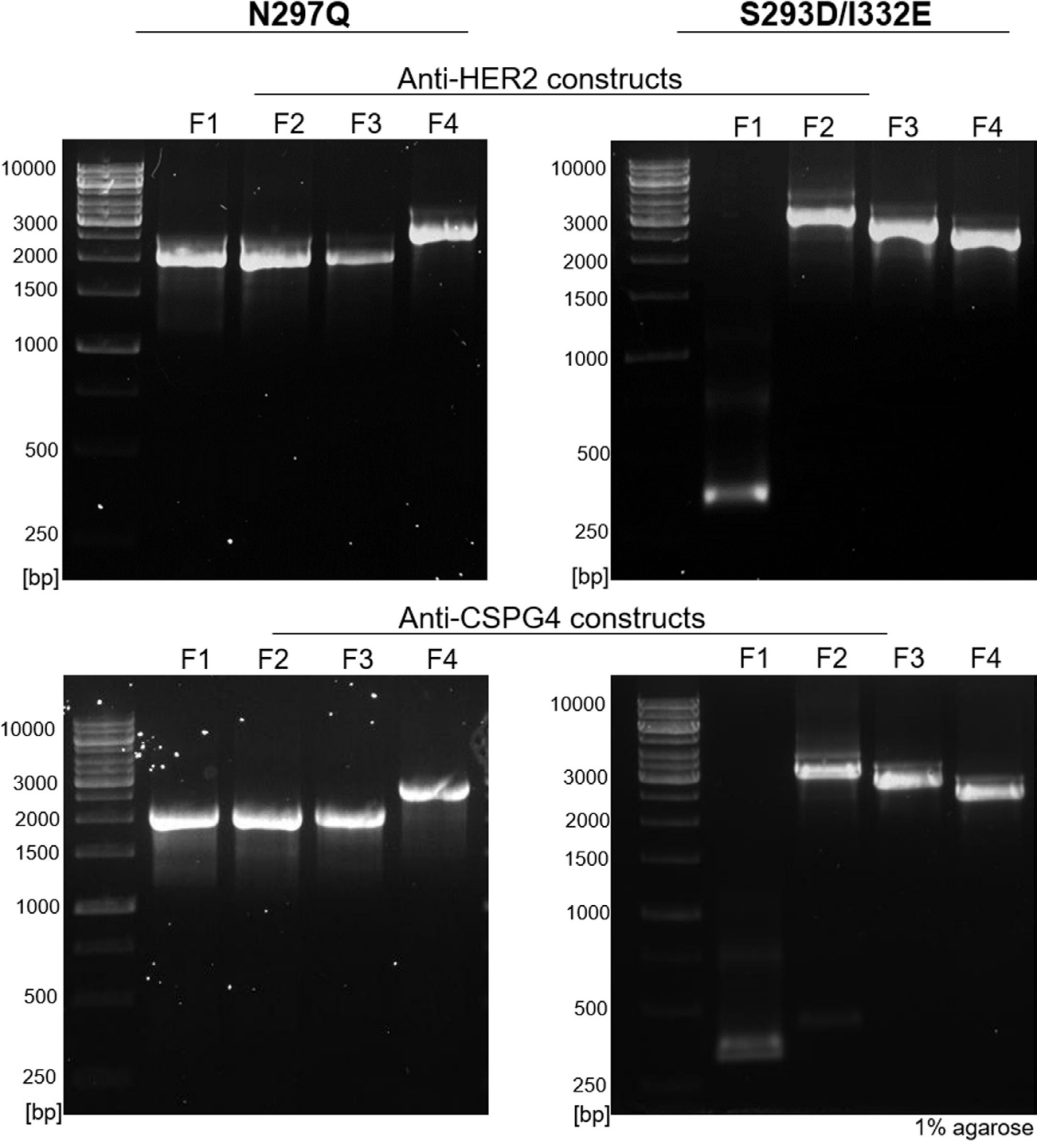
Agarose gel electrophoresis of polymerase incomplete primer extension (PIPE) PCR fragments for the generation of anti-HER2 and anti-CSPG4 N297Q (NQ) and S293D/I332E (DE) Fc mutant IgG1 antibodies. O’Generuler™ 1 kb DNA ladder (Thermo Fischer Scientific) was used as a molecular weight marker. The lanes labeled F1–4 represent the NQ and DE PIPE PCR fragments 1–4, depicted in Figure 1. The expected molecular weights (in bp) of each fragment are listed in Table 2.

Using our pipeline, we generated WT, N297Q (NQ), and S293D/I332E (DE) mutant antibodies recognizing two cancer antigens (i) the human epidermal growth factor receptor 2 (HER2/*neu*) (3), known to be overexpressed by 25% of breast carcinomas and (ii) the CSPG4, reported to be overexpressed in 70% of melanomas, a proportion of triple-negative breast cancers, and other solid tumors (41, 43, 44). In order to produce anti-HER2 IgG1 WT antibody, we used the variable regions of the 4D5-8 therapeutic antibody clone (trastuzumab) (2, 4, 45). The sequence was manually codon-optimized (Figure S1 in Supplementary Material), and cloned into the pVitro1-hygro-mcs vector (36). The anti-CSPG4 IgG1 WT construct was derived using the variable regions of a murine antibody (46) and cloned into pVitro1-hygro-mcs (36). The pVitro1 WT constructs were used as templates for the generation of the corresponding mutant variants. The DNA products of the mutagenic PIPE PCRs were visualized using agarose gel electrophoresis (Figure 2), confirming that the fragments were of the expected sizes.

All four mutant constructs were successfully cloned on first attempt and between 1 and 15 colonies were analyzed for the different constructs (Table S2 in Supplementary Material). The desired mutations were present in all the screened plasmids (each purified from a single colony) and no background contamination with the WT construct was observed. These findings suggest high mutagenesis efficiency for this cloning approach.

### Optimized Antibody Production with the Expi293F Expression System

**Figure 3:**
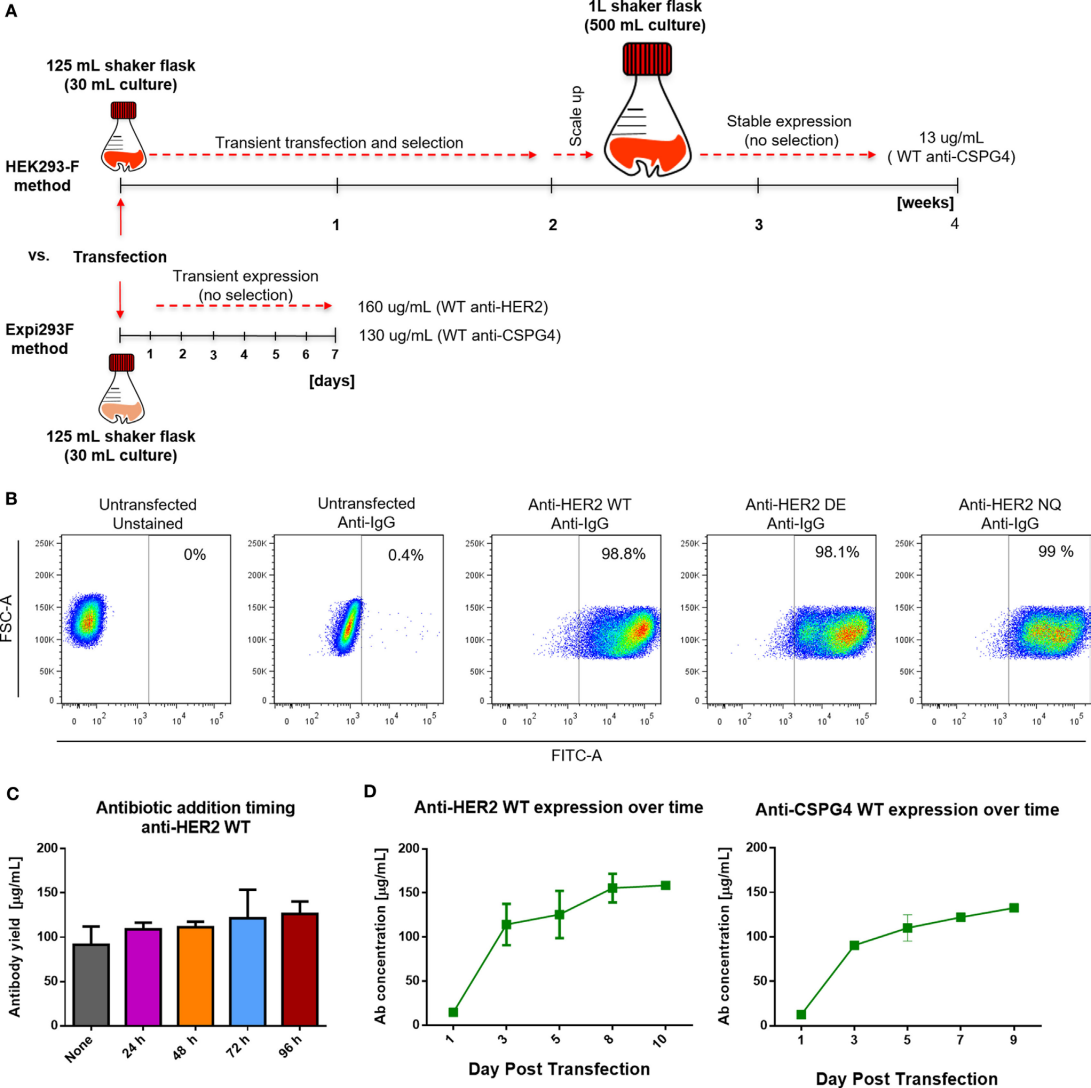
Cell transfection and antibody expression. **(A)** Schematic comparison of previously reported HEK293-F and the Expi293F expression systems with regard to required time, process, culture volumes, and antibody yields. **(B)** Flow cytometric dot plots depicting percentage of human IgG positive Expi293F cells 3 days post-transfection with anti-HER2 constructs. Cells were permeabilized and stained with anti-human IgG FITC; untransfected Expi293F cells (unstained and stained) were used as controls. **(C)** Effect of antibiotic selection addition at different times post Expi293F transfection with anti-HER2 wild-type (WT) construct—post-purification yields per mL of cell culture supernatant. Data from two independent experiments ± SD. **(D)** Concentration of WT anti-HER2 and anti-chondroitin sulfate proteoglycan 4 (CSPG4) antibodies per mL of cell culture supernatant over time measured by enzyme-linked immunosorbent assay. Data from two independent experiments ± SD.

We produced monoclonal antibodies both by using a previously described HEK293-F system as well as by employing the cloning approach described herein with the human embryonic kidney derivative cell line Expi293F. Antibody production with the previous method in HEK293-F cells was initiated using 30 mL shaker flask cultures under selection for 2 weeks, followed by a 2-week scale up to 500 mL shaker flask cultures. With the previous platform, after 4 weeks, we obtained an average of 13 µg/mL of anti-CSPG4 WT (by ELISA). By contrast, using the present transient expression Expi293F platform, we produced an average of 130 µg/mL anti-CSPG4 WT and 160 µg/mL anti-HER2 WT antibodies using only 30 mL shaker flask cultures volumes within 5–7 days post-transfection (detected by ELISA) (Figure 3A).

To evaluate the rate of transfection efficiency, we used flow cytometry to analyze the expression of IgG by permeabilized Expi293F cells 3 days after transfection with the anti-HER WT, DE, and NQ constructs (Figure 3B). A large proportion (≥98%) of transfected Expi293F cells was positive for human IgG, indicating a high transfection efficiency rate. To investigate whether the addition and timing of antibiotic selection (Hygromycin B) to Expi293F cell cultures could affect antibody yields, we expressed anti-HER2 WT antibody with or without the addition of Hygromycin B at different times following transfection and found that addition of Hygromycin B did not affect purified anti-HER2 antibody yields (Figure 3C). To then determine the optimal time to harvest the supernatants, we monitored the concentrations of anti-HER2 WT and anti-CSPG4 WT antibodies over time by ELISA. For both antibodies, the maximum production yields were reached between 5 and 7 days post-transfection (Figure 3D).

**Figure 4:**
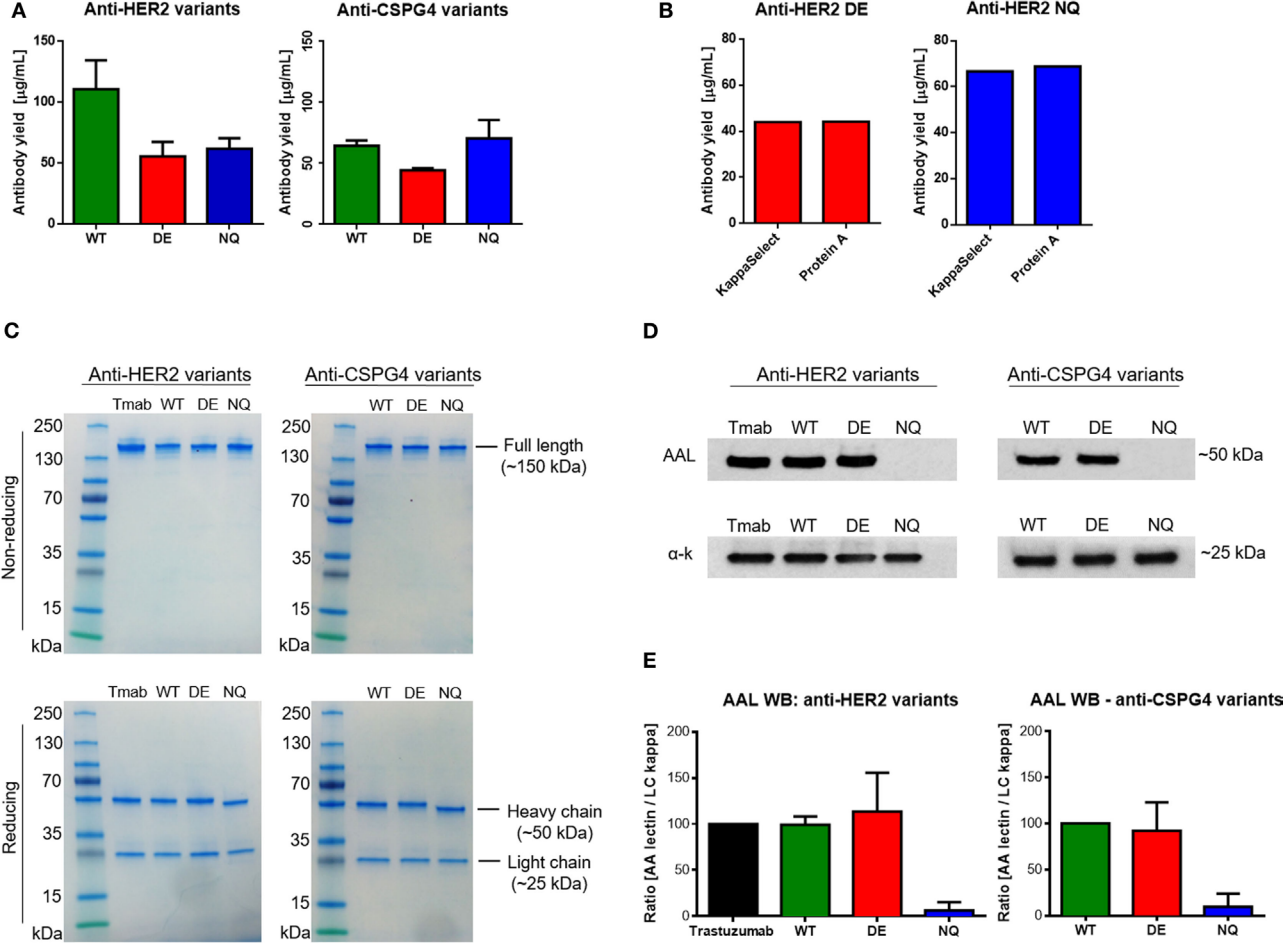
Purified antibody evaluations. **(A)** Wild–type (WT) and mutant anti-HER2 and anti-CSPG4 antibody yields per mL of cell culture supernatant after purification (data from two independent experiments ± SD). **(B)** Comparison of anti-HER2 mutant antibody yields after purification using HiTrap Kappa Select (GE Healthcare) or Protein A (Pierce™) columns. **(C)** Sodium dodecyl sulfate polyacrylamide gel electrophoresis visualization of purified anti-HER2 and anti-chondroitin sulfate proteoglycan (CSPG4) WT and Fc mutant antibodies under non-reducing (top) and reducing (bottom) conditions. PageRuler™ pre-stained protein ladder (Thermo Fischer Scientific) was used as molecular weight reference. Bands were visualized using InstantBlue™ protein stain (Expedeon). **(D)** Aleuria Aurantia Lectin (AAL) Western blot demonstrating fucosylation patterns of anti-HER2 (top left) and anti-CSPG4 (top right) Fc variants. Anti-human kappa chain antibody was used as a loading control (bottom panels). **(E)** Quantification of AAL Western blot signal using the ImageJ software. The AAL signal was normalized against the signal obtained with the anti-kappa chain antibody. The ImageJ peak area value of trastuzumab (Herceptin^®^, Roche) was considered a 100% and the peak area values of the other anti-HER2 variants were presented as a proportion of the first value. The values of anti-CSPG4 DE and NQ were calculated accordingly as a proportion of anti-CSPG4 WT. Data from three independent experiments ± SD.

We then measured total antibody yields by Expi293 cells after purification of anti-HER2 and anti-CSPG4 variants. We obtained an average of 56 µg/mL anti-HER2 DE, 62 µg/mL anti-HER2 NQ (Figure 4A, left), 44 µg/mL anti-CSPG4 DE, and 73 µg/mL anti-CSPG4 NQ (Figure 4A, right). We then confirmed that Fc mutations did not impair the binding of antibodies to protein A used for antibody purification: no significant differences in protein yields of either anti-HER2 DE (Figure 4B, left) or anti-HER2 NQ variants (Figure 4B, right) were measured after purification by protein A column, or by KappaSelect column, which binds antibody kappa light chains. We concluded that none of the mutations could alter antibody binding to either matrix and, thus, the purification method did not appear to influence antibody yields.

Next, the integrity and purity of different anti-HER2 and anti-CSPG4 antibody variants were confirmed through sodium dodecyl sulfate polyacrylamide gel electrophoresis (SDS-PAGE) under non-reducing (Figure 4C, top) and reducing (Figure 4C, bottom) conditions. All antibodies migrated according their expected approximate molecular weights of ~150 kDa (non-reducing conditions) and no other contaminating proteins or antibody fragments were observed. Under reducing conditions, the heavy chains of the WT and DE variants of both antibodies migrated according to the expected molecular weight of 50 kDa. Anti-HER2 and anti-CSPG4 NQ variants exhibited increased mobility compared with the WT and DE analogs, in accordance with previous reports (28). The light chains of all the variants were found to be of the expected size of 25 kDa.

To ascertain whether different Fc mutations influence glycosylation and to simultaneously evaluate the fucosylation patterns of the anti-HER2 and anti-CSPG4 variants, we probed purified antibody samples with AAL (fucose specific, Figure 4D). We used an anti-human kappa chain antibody as a loading control (Figure 4D, bottom strips). We observed no AAL signal with the anti-HER2 and anti-CSPG4 NQ variants, confirming that the introduced N297Q amino acid substitution has resulted in lack of core glycosylation (Figure 4D, top panel). We performed semi-quantitative analysis of the AAL Western blot data using the ImageJ software (Figure 4E). The values obtained with the NQ variants were close to background. No significant differences were observed between WT and DE mutant antibodies (both anti-HER2 and anti-CSPG4 variants). These findings suggest that mutating the Fc region of IgG1 at position 297 disrupts a conserved glycosylation site, most likely eliminating core glycosylation, while mutations at positions 293 and 332 do not affect the decoration of the core glycan with fucose.

Therefore, we confirmed the implementation of an optimized Expi293F expression platform with additional features and which outperforms that previously reported. Key attributes are five-fold: namely (i) provision for generation of mutant antibody variants; (ii) high mutagenesis efficiency; (iii) high transfection efficiency (eliminating the need for antibiotic selection); (iv) up to 10-fold higher antibody yields in ~17-fold smaller (30 mL) culture volumes; and (v) 3-fold reduced time frame (12 days) for implementation from concept to purified antibody (Table 3).

**Table 3:** Characteristics of the Expi293F vs. the HEK293-F cloning/expression platforms.

Expression host	Expi293F	HEK293-F
Wild-type (WT) antibody polymerase incomplete primer extension (PIPE) cloning	Yes	Yes
Generation of Fc mutants	Yes	No
Expression culture conditions	Serum free	10% FBS
Expression system type	Transient	Stable
Antibody concentration (sup)	130 µg/mL (anti-CSPG4 WT)	13 µg/mL (anti-CSPG4 WT)
Working culture volume (expression)	30 mL	500 mL
Time required for cloning/sequencing verification	3 days	3 days
Time required for large scale plasmid DNA production/transfection	2 days	2 days
Time required for expression/purification	1 week	4 weeks
Total time required (whole platform)	12 days	33 days

### Fc Mutants Retain Fab-Mediated Binding to Antigens but Exhibit Differential Fc-Mediated Receptor Recognition Compared with WT Antibodies

**Figure 5:**
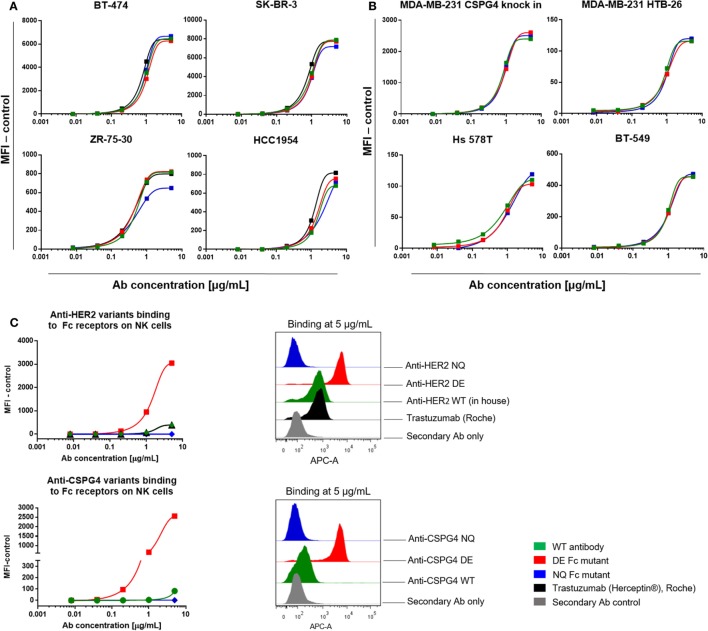
Antibody target antigen and Fc receptors (FcRs) binding evaluations. **(A)** Target antigen recognition of anti-HER2 Fc variants on HER2-overexpressing cancer cell lines. All anti-HER2 Fc variants including the positive control trastuzumab (Herceptin^®^, Roche) bound to the target antigen in a similar dose-dependent manner. **(B)** Target recognition of anti-chondroitin sulfate proteoglycan 4 (CSPG4) Fc variants on triple-negative breast cancer cell lines. All the anti-CSPG4 Fc variants bound to the target antigen in a similar dose-dependent manner. **(C)** Left: binding of anti-HER2 (top) and anti-CSPG4 (bottom) Fc variants to FcγRIII on fresh human peripheral blood NK cells with increasing antibody concentrations (0.008–5 µg/mL). Right: flow cytometric histograms depicting antibody binding at saturating concentrations (5 µg/mL). Graphs are representative of two independent experiments; data were normalized against an isotype control **(A,B)** or secondary antibody control **(C)**.

We next investigated the target antigen recognition and binding properties of engineered antibody variants against different cancer cell lines expressing cell surface HER2 (Figure 5A; Figure S2 in Supplementary Material) or CSPG4 (Figure 5B). All HER2 variants recognized HER2 on BT-474 (top left), SK-BR-3 (top right), ZR-75-30 (bottom left) and HCC1954 (bottom right) breast cancer cell lines, and bound in a dose-dependent manner and with comparable kinetics to the commercially produced trastuzumab (Herceptin^®^, Roche). Similarly, for the engineered antibodies recognizing CSPG4 (Figure 5B), no differences were observed in their binding characteristics to the CSPG4-overexpressing CSPG4 knock-in MDA-MB-231 (top left), and natural lower-expressing MDA-MB-231, HTB-26 (top right), Hs 578T (bottom left), and BT549 (bottom right) breast carcinoma cell lines.

We then investigated the binding characteristics of anti-HER2 and anti-CSPG4 Fc variants to FcγRIII, the Fcγ receptor expressed by NK cells (47) (Figure 5C). Fluorescence intensities observed for human peripheral blood NK cells incubated with anti-HER2 (top) and anti-CSPG4 (bottom) NQ variants were similar to those measured for the secondary anti-human kappa chain APC-conjugated antibody control at all concentrations tested, suggesting that the NQ mutation resulted in impaired binding to FcγRIII. Binding of the WT anti-HER2 antibodies, including the commercially available trastuzumab (Herceptin^®^) to human NK cells were detected only at the highest concentrations tested (1 and 5 µg/mL). Similarly, the anti-CSPG4 WT antibody only bound to NK cells at high (5 µg/mL) concentrations. By contrast, the anti-HER2 and anti-CSPG4 DE variants displayed up to 7-fold (anti-HER2) and 17-fold (anti-CSPG4) higher binding to FcγRIII on NK cells compared with binding of WT antibodies at 5 µg/mL concentrations to FcγRIII (Figure 5C).

These findings suggest that while Fc-mutated antibodies retain Fab-mediated recognition of target antigen-expressing cancer cells, DE mutant antibodies have enhanced, and NQ mutants have diminished binding to FcγRIII on human primary NK cells compared with their WT equivalents.

### DE Mutant Antibodies Have Enhanced, While NQ Mutants Have Diminished Ability to Induce Fc-Mediated Activation of NK Cells Compared with WT Antibodies

**Figure 6:**
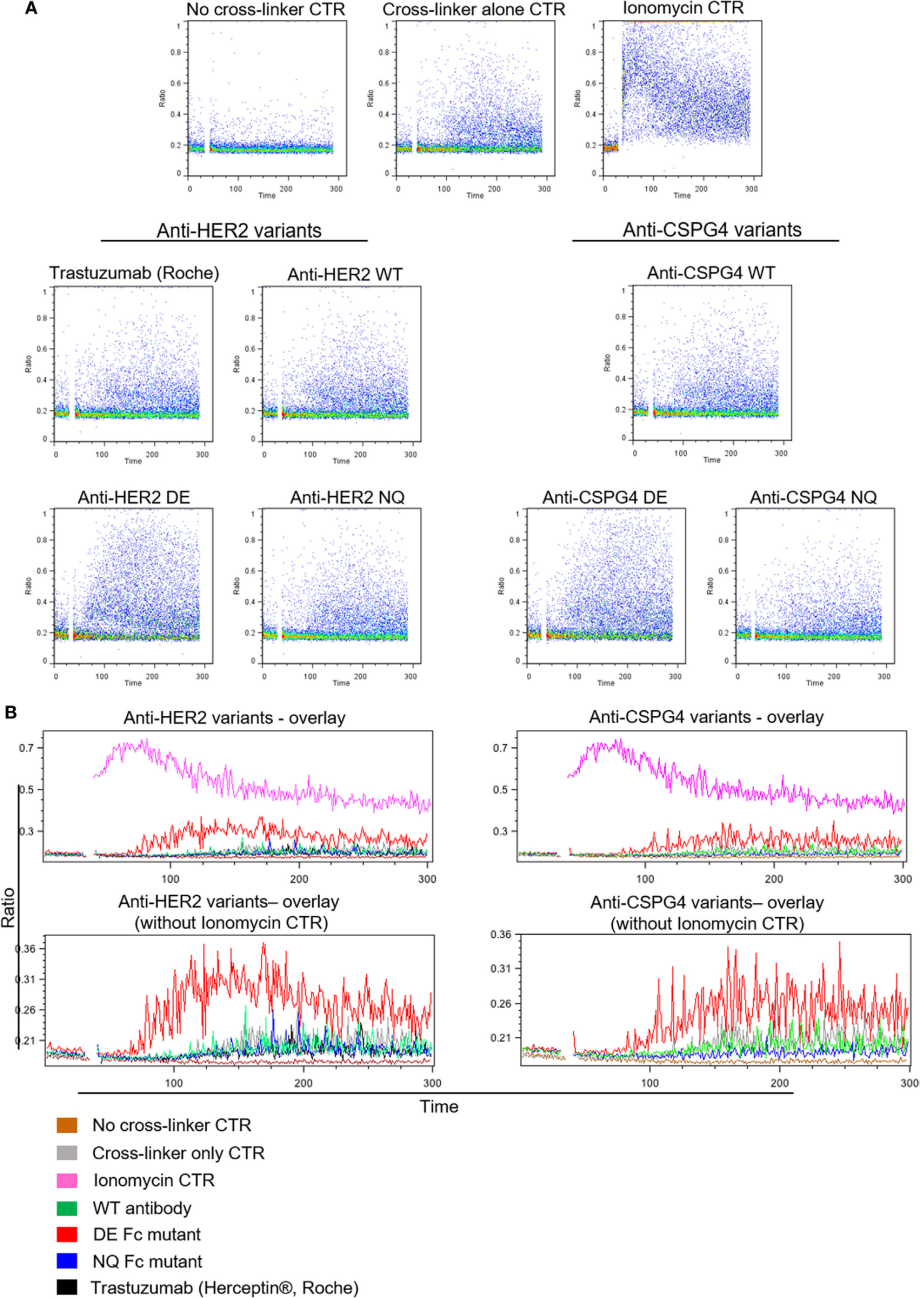
Antibody Fc-mediated calcium mobilization of human NK cells. **(A)** Flow cytometric dot plot graphs of Ca^++^ flux assay measurements showing activation of NK cells pre-incubated with different anti-HER2 Fc (left) and anti-chondroitin sulfate proteoglycan 4 (CSPG4) (right) Fc variants after cross-linking with a polyclonal anti-IgG antibody. Ca^++^ flux into the cells was visualized through the increase in the DAPI/Indo-1 (blue) ratio over time. **(B)** Histogram overlay demonstrating the differences in Ca^++^ influx between different anti-HER2 (left) and anti-CSPG4 (right) antibody variants depicted as the changes in the DAPI/Indo-1 Blue fluorescence ratio over time. The top overlays include all antibody variants and controls and the bottom exclude the ionomycin control to more clearly demonstrate the differences between the antibody variants on a smaller scale. Data representative of three independent experiments.

To further assess the ability of antibody variants to engender NK effector cell activation through engagement of FcγRIII, we measured calcium ion influx by cross-linking with polyclonal anti-IgG antibodies to mimic immune complex formation. No calcium flux was detected in cells not treated with cross-linker (Figure 6A, top). The levels of calcium flux following cross-linking of WT antibodies [anti-HER2 WT, trastuzumab (Herceptin^®^), anti-CSPG4 WT] and NQ antibodies (anti-HER2 NQ and anti-CSPG4 NQ) were all comparable to background levels of control samples given cross-linker only to engage endogenous IgG (Figure 6). On the other hand, cross-linking of either anti-HER2 DE or anti-CSPG4 DE antibody variants triggered higher intracellular calcium mobilization compared with all other antibodies (Figure 6). These findings, confirmed with NK cells from different human donors (Figure S3 in Supplementary Material), are consistent with the higher binding attributes of DE mutant variants to FcRIII on NK cells (Figure 5C).

### Anti-HER2 Variants Retain Their Ability to Inhibit HER2-Overexpressing Cancer Cell Proliferation but Differ in Their Potency to Trigger NK Cell-Mediated Anti-Tumor Functions

**Figure 7:**
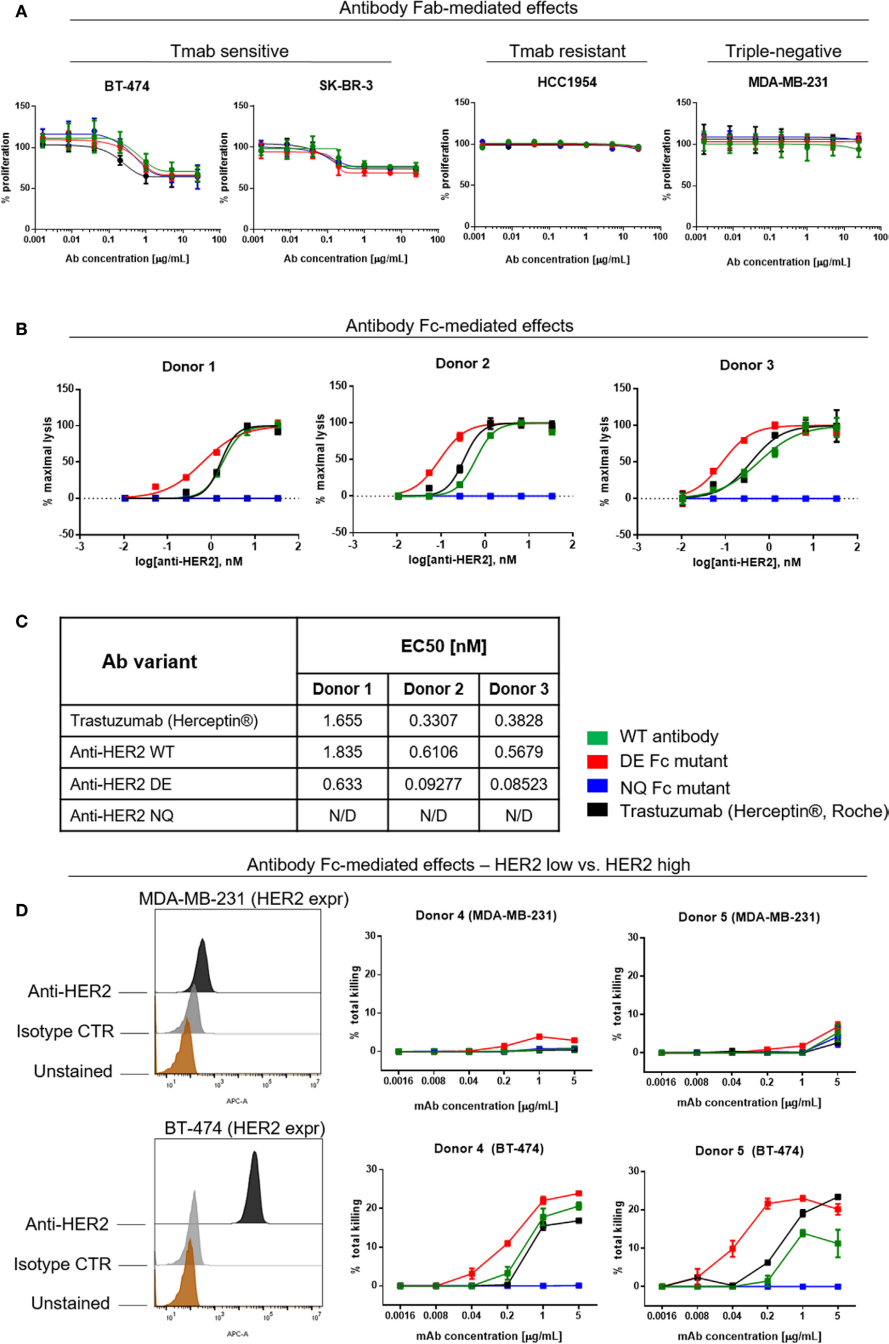
Assessments of direct and Fc-mediated effects of anti-HER2 Fc variants against breast cancer cells. **(A)** Effects of anti-HER2 antibody variants on the proliferation of trastuzumab-sensitive (BT-474, SK-BR-3), trastuzumab-resistant (HCC1954) and triple-negative (MDA-MB-231) breast cancer cell lines. Anti-HER2 variants inhibited the proliferation of BT-474 and SK-BR-3 cells in a similar dose-dependent manner, but did not affect the proliferation of MDA-MB-231 or HCC1954 cells. Graphs represent an average of two experiments ± SD. **(B)** Human peripheral blood NK cell-mediated ADCC of BT-474 cancer cells induced by anti-HER2 variants measured by LDH release. Graphs are representative of independent experiments with three different human NK cell donors; data were normalized to minimal and maximal cell lysis. Error bars represent SEM values from technical replicates. N/D: not detected. **(C)** Effective concentration [(EC50) nM] measurements of ADCC by three human NK cell donors. **(D)** NK cell-mediated ADCC (measured by LDH release) of HER2 low (MDA-MB-231) and HER2 high (BT-474) breast cancer cells induced by anti-HER2 variants. The flow cytometric histograms on the left depict HER2 expression levels in MDA-MB-231 (top) and BT-474 (bottom) compared to unstained cells or cells stained with isotype control mAb. The graphs represent total cell killing levels of MDA-MB-231 cells (top) and BT-474 cells (bottom) mediated by NK cells from two different donors (Donors 4 and 5) at different concentrations of anti-HER2 variants.

Trastuzumab (Herceptin^®^) has been reported to directly inhibit the proliferation of HER2-over-expressing cell lines (1, 2, 4, 48). We, therefore, examined whether these attributes are retained by the anti-HER2 Fc variants. WT and mutant anti-HER inhibited the proliferation of the HER2-overexpressing BT-474 and SK-BR-3 breast cancer cells in a dose-dependent manner, and with comparable potency to those of the clinically available trastuzumab (Herceptin^®^). None of the variants could reduce the proliferation of the low HER2-expressing triple-negative MDA-MB-231 or of the trastuzumab-resistant HCC1954 breast cancer cell lines (Figure 7A) (49). We, therefore, concluded that Fc-engineered antibodies can retain the direct Fab-mediated effects of the original unmodified clone.

A known anti-tumor mechanism of action of trastuzumab (Herceptin^®^) is the ability to trigger antibody-dependent cellular cytotoxicity (ADCC) of HER2-expressing cancer cells mediated by NK cells (11, 50). Therefore, we studied the potency of different anti-HER2 Fc variants to induce tumor cell cytotoxicity (ADCC) of the HER2-overexpressing BT-474 cells (target cells) mediated by fresh peripheral blood NK cells from three different donors (effector cells) (Figure 7B) (51–53). The in-house produced anti-HER2 WT antibody and trastuzumab (Herceptin^®^) showed similar tumor cell killing potencies mediated by NK cells from all three human donors. Anti-HER2 DE induced higher tumor cell killing at significantly lower concentrations than WT antibodies. All antibodies showed similar levels of ADCC at saturating concentrations (5 µg/mL). In line with diminished binding to FcγRIII on the surface of NK cells, the anti-HER2 NQ mutant IgG1 failed to induce any tumor cell killing above controls (EC50 of variants for each independent experiment, Figure 7C).

Furthermore, we tested the potency of anti-HER2 variants to trigger NK cell-mediated ADCC against cancer cells that express low levels of the target. We chose the triple-negative breast cancer cell line MDA-MB-231 known to express very low levels of HER2/*neu* (Figure 7D), and the HER2-overexpressing cell line BT-474 to compare the effects of the target expression on ADCC levels induced by NK cells from the same donors (Figure 7D, Donors 4 and 5). We observed no or very low ADCC against MDA-MB-231 with all the variants (Figure 7, top panels), while against BT-474, anti-HER2 DE, WT, and trastuzumab induced dose-dependent tumor cell lysis (Figure 7D, bottom panels), consistent with the findings depicted in Figure 7B.

Therefore, while antibody Fab-mediated direct effects are not affected, Fc-modified antibody variant engineering allows the manipulation of antibody binding kinetics to effector cells, and can alter antibody functional capacity and potency to mediate immune effector cell activation and NK cell-mediated cytotoxicity of tumor cells.

## Discussion

With monoclonal antibody therapies firmly established as part of the standard care of treatment for solid tumors and hematological malignancies (54–56), designing antibodies to engender well-defined Fab-mediated and Fc-mediated anti-cancer properties is highly desirable. Specifically, Fc-mediated effects can have a major impact on the ability of antibodies to engage cells of the immune system and can influence their anti-tumor functions and efficacy.

Here, we designed and implemented a novel seamless cloning and expression platform for the generation of a panel of anti-cancer monoclonal antibodies with modified human Fc regions. We evaluated the functional impact of antibody engineering on antibody Fab- and Fc-mediated functions. This strategy allows the generation of WT and Fc mutant antibodies of any specificity at high mutagenesis and transfection efficiency, short time frames for cloning and production, and high yields of pure material. Compared to WT antibodies produced using a previous cloning approach, this represents 10-fold increased yields at a 3-fold reduced time frame of 12 days from design to purified antibody material (Table 3). This approach permits the generation of Fc-modified variants of any antibody clone and may find wide applications in antibody engineering and functional screening.

Compared with existing mutagenesis cloning methods, our approach is unique in combining the following features: (i) high efficiency; (ii) no need of restriction or ligation enzymes; (iii) requirement for only a single step PCR; (iv) designed for mutagenesis/cloning of large expression ready plasmid constructs; (v) employs universal primers which could be applied to any IgG1 antibody construct; and (vi) applicability for the generation of different versions of the same antibody construct (36, 37, 40, 57). These features, combined with rapid, small volume, and high yield transient expression in mammalian Expi293F cells constitute a unique and novel methodology which can facilitate selection of therapeutic antibody candidates with the most suitable attributes by employing mechanistic and potency evaluations.

While all antibody variants retained recognition of target antigens expressed on tumor cells, engagement of FcγRs on human primary NK cells was affected in Fc-mutated variants. The DE and NQ mutants showed enhanced and diminished binding properties, respectively, compared with binding of WT antibodies, consistent with previous findings (58). Here, binding studies of the Fc variants were conducted by flow cytometry on natively expressed FcRs and in the presence of endogenous immunoglobulins on the surface of fresh human NK cells. Hence, our 7- to 17-fold increased affinity of the DE variants compared with native antibodies may underestimate absolute increased affinities, previously measured with recombinant FcγRIIIa (58). Furthermore, although we engineered our antibody panels for each clone to be of the same isotype, with identical Fc region sequences, we observed that DE mutations improved the binding of anti-CSPG4 to NK cells more than the same mutations improved the binding of anti-HER2 enhanced DE variant. It is possible that these differences may depend on the antibody Fab regions and that mutating Fc regions in an identical way could still result in different Fc-mediated properties for different antibodies and merits further study. We also found differences in enhanced binding between DE and WT antibodies with different donors. It is, therefore, possible that enhanced kinetics would also be subject to donor-dependent effects and this requires investigation.

When we interrogated antibody Fc-mediated NK cell activation, we found enhanced calcium mobilization by mutant antibodies with high FcR-binding attributes. Furthermore, we observed low levels of Calcium influx triggered by endogenous IgGs on the surface of NK cells, which did not increase after the cells were incubated with WT antibodies. This may represent a picture close to the physiological conditions, where WT therapeutic antibodies would have to compete with endogenous IgGs with similar affinities for their cognate Fcγ receptors. This may be addressed through increasing the affinity of therapeutic antibodies to CD16 (59).

We demonstrated that while all anti-HER2 antibody variants retain their Fab-mediated direct effects in restricting HER2-expressing breast cancer cell proliferation, Fc-modified anti-HER2 antibodies engender differential anti-tumor cell cytotoxicity potencies by human NK cell effectors. Fc engineering may, therefore, provide a means of manipulating antibody binding kinetics to effector cells, and can alter the antibody’s functional capacity and potency to mediate immune effector cell activation and cell-mediated cytotoxicity.

Even though there are conflicting reports about the ability of trastuzumab to trigger NK cell-mediated killing in low HER2-expressing cancer cells, in this study, we observed very low ADCC activity of anti-HER2 variants against cancer cells expressing low levels of the target antigen (51, 60). Although this may indicate the likely inability of Fc engineered antibodies to target low tumor antigen-expressing cancer cells, these findings could also be interpreted as favorable in terms of potential safety of clinical application of Fc-optimized anti-HER2 antibodies. By targeting high-expressing tumor cells but not low HER2-expressing normal tissues, such as cardiomyocytes, their application may engender anti-tumor effects in the absence of on-target, off-tumor toxicities (61).

We confirmed that our generated N297Q mutants completely lack fucosylation. The N297Q mutation most likely results in loss of the core glycan, previously reported to alter the “open” conformation of WT IgG1 antibodies in favor of a “closed” confirmation, leading to impaired ability to engage FcRs (26). On the other hand, loss of fucose from the N-linked core glycan can substantially improve IgG binding to FcγRIIIA and ADCC. Therefore, differences in the glycosylation of optimized Fc mutants may contribute to their ability to outperform their WT counterparts in FcR engagement and effector cell activation (62). In this study, we demonstrate that the enhanced FcR and Fc-mediated functional attributes of the S293D/I332E mutants are not linked to differential fucosylation. The exact mechanisms behind this functional superiority are currently unknown; unraveling these would require further in-depth structural, interaction, and functional studies.

The importance of engineering monoclonal antibodies with defined Fc functions is highlighted by findings of reduced clinical responses to monoclonal antibodies, such as cetuximab (anti-EGFR), trastuzumab (anti-HER2), and rituximab (anti-CD20), in individuals who carry FcR polymorphisms that alter antibody binding affinities to immune effector cells (63–67). Different approaches have been developed to enhance the potency of monoclonal antibodies in activating effector cells against cancer (21). Optimization of IgG Fc domains through introduction of point mutations or glyco-engineering can influence binding kinetics to FcR. Fc mutant versions of trastuzumab (anti-HER2), alemtuzumab (anti-CD52), rituximab (anti-CD20), and others have shown significantly improved binding to FcγRs and enhanced effector functions (58, 68). Since removal of fucose from the *N*-linked core glycan can dramatically improve IgG–FcγRIIIA interactions and ADCC of tumor cells (62), two afucosylated antibodies (obinutuzumab and mogamulizumab) are approved for clinical use in hematological oncology (69, 70). An important reported advantage of certain Fc-optimized therapeutic antibodies is that they exhibit enhanced binding to FcγRIIIA even in the presence of polymorphisms that otherwise lower affinity for IgG (58, 64). In this aspect, a future development of our platform could include functional studies of the capacity of Fc-optimized variants to activate NK cells with defined FcγRIIIA genotype. Specific Fc mutations can also improve antibody pharmacokinetic characteristics through improved binding to the neonatal FcR, FcRn (21). Experimental approaches also entail switching the antibody isotype from the traditionally preferred IgG (mainly IgG1) to other isotypes, such as IgA or IgE, which could also provide advantages through alternative immune surveillance properties in specific tissues and through activating immune effector cell types that may be different to those engaged by IgG (18, 45, 71–74).

In this study, we also generated IgG1 antibody variants with vastly reduced FcR engagement and with impaired effector functions. Abrogation of antibody binding to FcRs and abolition of Fc-mediated effector functions is a useful approach for the design of antibodies with direct agonistic or antagonistic activities, antibody–drug conjugates or checkpoint blockade inhibitor antibodies. In some cases, effector cell engagement may not always be desirable, may impede Fab-mediated effects or bear safety hazards if intact Fc-mediated effector functions are retained (22, 23, 25, 75). Antibodies of the IgG2 or IgG4 isotypes are traditional choices in such drug design strategies. Nevertheless, IgG2 and IgG4 antibodies may not completely lack effector cell engagement (76). Furthermore, the latter may retain the potential to interact with other IgG Fc domains or undergo Fab arm exchange (FAE) which can lead to reduced activity or off-target effects (24). Efforts have, therefore, focused toward abolishing Fc-mediated functions to avoid potentially dangerous immune-mediated toxicities. In addition, monoclonal antibodies used for cancer therapy are often associated with a range of adverse reactions including triggering cytokine storm or hypersensitivity responses (22, 23). Where the purpose of the therapy is to engender Fab-mediated functions (25), complete abrogation of Fc effector capabilities could often be a necessity for certain antibody therapeutic design applications.

Monoclonal antibodies of the same immunoglobulin isotype recognizing different cancer antigens often act through different immune-mediated mechanisms. The same Fc-modifications may engender different effector properties by antibodies with variable specificities and affinities to different antigens or antigenic epitopes. In this context, our approach provides a convenient tool to enable investigation of the effects of Fc point mutations on the function of monoclonal antibodies of any specificity. To exemplify this, we quickly generated Fc variants of monoclonal antibodies against two tumor-associated antigens, with which we were able to delineate antibody Fab-mediated and Fc-mediated mechanisms of action. This demonstrated the capability of the system to enable control of effector functions through introduction of point mutation capabilities. The universal nature of the Fc modification features built into our approach opens the door toward the generation of a range of Fc variants of antibodies of any specificity. Furthermore, serum-free production and quick high antibody yields could provide sufficient high quality material to facilitate early *in vitro* and *in vivo* screening, downstream translation to good manufacturing practice (GMP) pathways and facilitate potential clinical application.

In summary, with this design strategy, we are able to manipulate anti-cancer antibody Fc regions. We delineate the binding and functional attributes of WT and Fc-modified agents and evaluate their Fab-mediated and Fc-mediated functions and potency. This approach can find broad application in therapeutic antibody engineering and translation for cancer therapy and potentially in other areas beyond the cancer immunotherapy field.

## Ethics Statement

Blood cone samples from anonymized donors were purchased from the National Health Service Blood and Transplant Service, United Kingdom. Sample processing was supported through a local ethical framework conducted in accordance with the Helsinki Declaration and approved by the NHS Research Ethics Committee (Van Cutsem et al.), Guy’s and St. Thomas’ NHS Trust (“Immunopathogenesis and Molecular Biology in Breast Cancer Subtypes,” REC reference number: 13-LO-1248).

## Author Contributions

KI, SK, and AT conceived and designed the study. KI, JF-S, TD, SM, SC, PK, and IC helped with the development of the methodology. KI, JF-S, DA, TD, SM, SC, HB, AC, PK, and IC acquired the data or helped with the data analysis and interpretation. KI, JF-S, DA, SM, SC, HB, PK, MF, RM, DJ, AB, JM, JS, EJ-J, AT, and SK discussed and interpreted the data and edited the manuscript. SK and AT supervised the study. SK led and coordinated the project. KI and SK wrote the manuscript.

## Conflict of Interest Statement

SK and JS are founders and shareholders of IGEM Therapeutics Ltd. All other authors declare not conflicts of interest.
