# Supplementary material for: Functionally Active Fc Mutant Antibodies Recognizing Cancer Antigens Generated Rapidly at High Yields
Source: Front Immunol. 2017 Sep 11;8:1112. doi: 10.3389/fimmu.2017.01112 (PMC5604060; doi:10.3389/fimmu.2017.01112)
Supplement: Supplementary file 1 [file Data_Sheet_1.DOCX]

**Supplemental Files**

Anti-HER2 heavy chain variable fragment – codon optimized, GC content < 55%

gaggtgcagctggtggagtctggaggcggactggtgcagcctggcggatctctgagactg

E V Q L V E S G G G L V Q P G G S L R L

tcttgtgccgcctctggatttaatattaaggatacatatattcactgggtgagacaggcc

S C A A S G F N I K D T Y I H W V R Q A

cctggcaagggactggagtgggtggccagaatctatcctacaaacggatatactagatac

P G K G L E W V A R I Y P T N G Y T R Y

gctgattctgtgaagggaagatttaccatctctgccgatacttctaagaataccgcctat

A D S V K G R F T I S A D T S K N T A Y

ctgcagatgaactctctgcgggccgaggataccgccgtgtattattgttctcggtggggc

L Q M N S L R A E D T A V Y Y C S R W G

ggcgacggcttttacgccatggattattggggccagggcactctggtgaccgtgtcctca

G D G F Y A M D Y W G Q G T L V T V S S

Anti-HER2 light chain variable fragment – codon optimized, GC content < 53%

gacatccagatgacccagtctccctcctctctgtctgcctctgtgggcgatcgggtgacc

D I Q M T Q S P S S L S A S V G D R V T

attacctgtcgggcctctcaggatgtgaataccgccgtggcctggtatcagcagaagcct

I T C R A S Q D V N T A V A W Y Q Q K P

ggcaaggcccctaagctgctgatttattctgcctcttttctgtattctggcgtgccttct

G K A P K L L I Y S A S F L Y S G V P S

cggttttctggctctcggtctggcactgattttactctgactatttcttctctgcagcct

R F S G S R S G T D F T L T I S S L Q P

gaggattttgccacttattattgtcagcagcactatactactcctcctacttttggccag

E D F A T Y Y C Q Q H Y T T P P T F G Q

ggcaccaaagtggagattaaac

G T K V E I K

10000

3000

1500

1000

500

**Anti-HER2 WT**

pVitro 1

pVitro 2

VH

VL


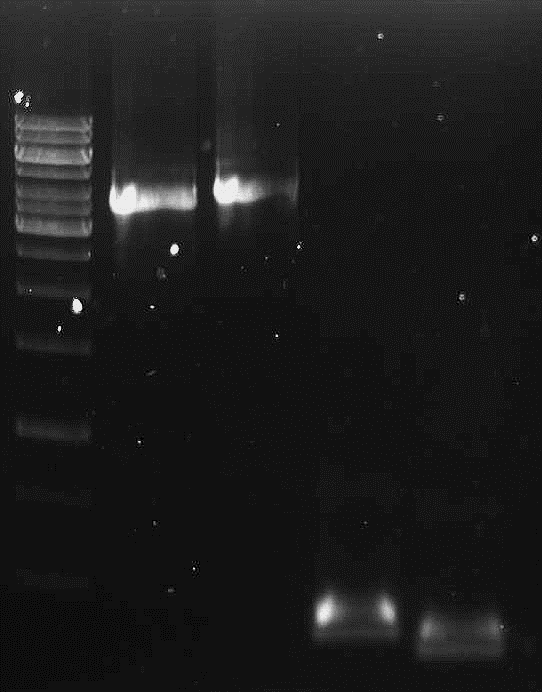


**B. C.**

**Supplemental Figure 1: A.** Codon optimized nucleotide and amino acid sequences of the variable fragments of anti-HER2 antibody based on the amino acid sequence of trastuzumab obtained from the DrugBank database. **B.** Agarose gel electrophoresis of PIPE DNA fragments for the generation of wild type anti-HER2 WT IgG1; **C.** Effect of codon optimization on antibody yields of anti-HER2 wild type antibody.

**
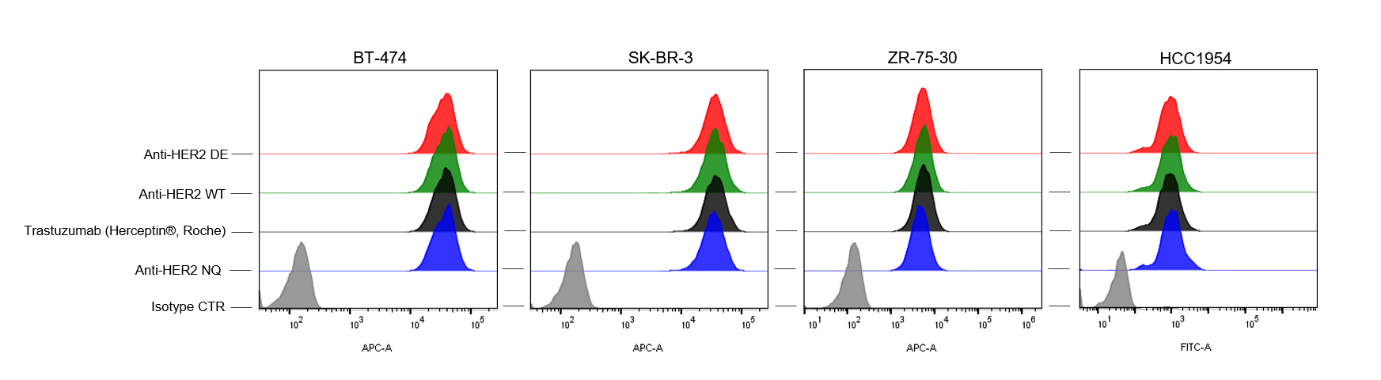
**

**Supplemental Figure 2:** Flow cytometric histograms depicting the binding of anti-HER2 variants to BT-474, SK-BR-3, ZR-75-30 and HCC1954 cancer cell lines at 5 μg/mL. No differences were observed in the binding of different anti-HER2 variants to the cells.


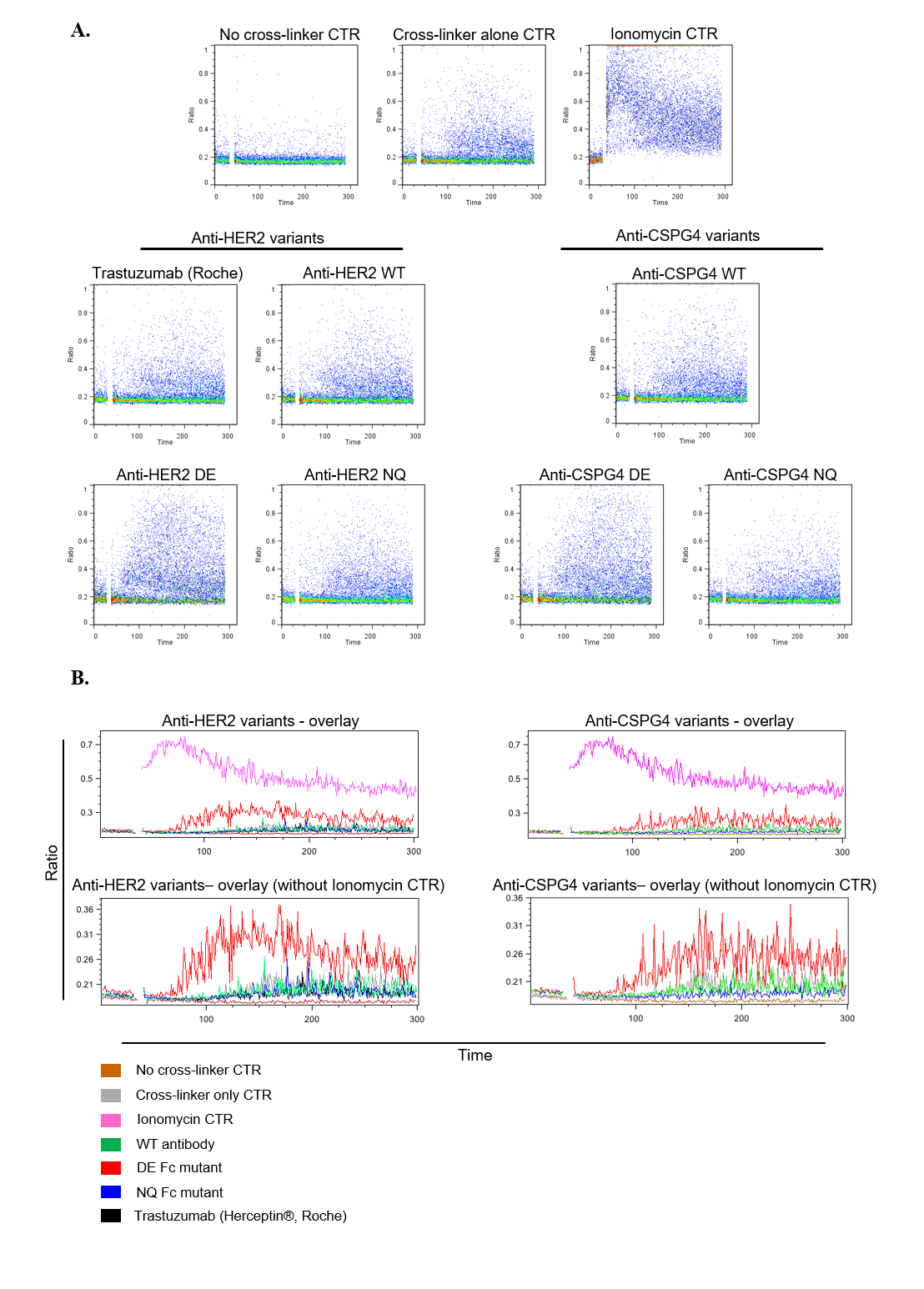


**Supplemental Figure 3:** Antibody Fc-mediated calcium mobilisation of human NK cells. **A.** Flow cytometric dot plot graphs of Ca^++^ flux assay measurements showing activation of NK cells pre-incubated with different anti-HER2 Fc (left) and anti–CSPG4 (right) Fc variants after cross-linking with a polyclonal anti-IgG antibody. Ca^++^ flux into the cells was visualized through the increase in the DAPI/Indo-1 (blue) ratio over time. **B.** Histogram overlay demonstrating the differences in Ca^++^ influx between different anti-HER2 (left) and anti–CSPG4 (right) antibody variants depicted as the changes in the DAPI/Indo-1 Blue fluorescence ratio over time. The top overlays include all antibody variants and controls and the bottom exclude the Ionomycin control to more clearly demonstrate the differences between the antibody variants on a smaller scale. Data representative of three independent experiments.

**Supplemental Table 1:** PCR protocol used for the execution of PIPE PCR.

| Reagent | Quantity |
| --- | --- |
| **DNA template** | 10 ng |
| **5` primer** | 25 pmol |
| **3` primer** | 25 pmol |
| **Phusion Flash Mastermix** | 25 μL |
| **ddH_2_0 (DNase free)** | up to 50 μL |

**Supplemental Table 2:** PIPE cloning/mutagenesis outcome for different antibody variants

| Construct | Number of colonies | Screened colonies | Desired mutations present |
| --- | --- | --- | --- |
| **Anti-HER2 NQ** | 12 | 6 | all |
| **Anti-HER2 DE** | 3 | 3 | all |
| **Anti-CSPG4 NQ** | 6 | 6 | all |
| **Anti-CSPG4 DE** | 1 | 1 | all |
